# Account of Diseases in an Eastern District of London, from April 20, to May 20, 1804

**Published:** 1804-06-01

**Authors:** 


### *Account of Diseases in an Eastern District of London, from April 20, to May 20, 1804.*

| | |
|---|---|
| **ACUTE DISEASES.** | |
| Peripneumonia | 2 |
| Catarrhus | 5 |
| Measles | 3 |
| Rheumatismus Acutus | 4 |
| **CHRONIC DISEASES.** | |
| Tussis | 13 |
| Tussis cum Dyspnœa | 17 |
| Hydrops Pectoris | 4 |
| Pleurodyne | 5 |
| Anasarca | 5 |
| Ascites | 4 |
| Ophthalmia | 3 |
| Fluor Albus | 14 |
| Menorrhagia | 12 |
| Chlorosis | 5 |
| Amenorrhœa | 7 |
| Dyspepsia | 5 |
| Vomitus | 2 |
| Procidentia Vaginæ | 1 |
| Herpes | 4 |
| Rheumatismus Chronicus | 13 |
| **PUERPERAL DISEASES.** | |
| Peritonitis | 3 |
| Menorrhagia Lochialis | 5 |
| Rhagus Papillæ | 4 |
| Mastodynia | 6 |
| **INFANTILE DISEASES.** | |
| Aphthæ | 3 |
| Convulsio | 2 |
| Herpes | 2 |
| Vermes | 4 |

The present state of disease does not offer any thing new or very interesting to the medical inquirer. Catarrhs and other affections of the chest still continue. These, which generally constitute a large proportion of the disorders occurring in the winter, or in the earlier months of the spring, have been continued later than usual in the present season. This has proved a discouraging circumstance to some who are subject to an annual return of these complaints, but who generally observe them to retire at an earlier period of the year, and are therefore ready to infer, that instead of being periodical, their complaints will become continual. Their continuance, however, may be ascribed to the very frequent and sudden changes of temperature during the period referred to. The degree of heat prevailing in the early months of the spring, induced some persons to expose themselves to the danger of catching cold, either by a change in their clothing, or by placing themselves in a current of air, which, however pleasant and refreshing, has been the occasion of producing a relapse, or protracting their disease.

